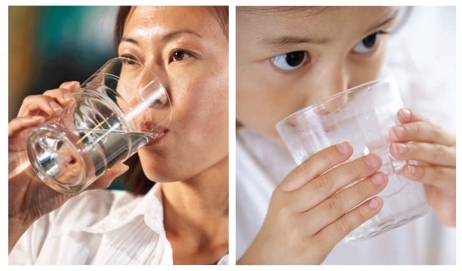# Exposure on Tap: Drinking Water as an Overlooked Source of Lead

**DOI:** 10.1289/ehp.118-a68

**Published:** 2010-02

**Authors:** Rebecca Renner

**Affiliations:** **Rebecca Renner**, PhD, of Williamsport, Pennsylvania, is a long-time contributor to *EHP* and *Environmental Science & Technology*. Her work has also appeared in *Scientific American, Science*, and Salon.com

Providence, Rhode Island, and Portland, Oregon, are two cities that by all accounts have well-run water utilities and health departments. Both have also had recurring problems with lead in tap water, yet both—according to some critics—have downplayed the potential importance of lead in tap water as a route of exposure. The experiences of these cities and others across the United States illustrate the difficulty not only of determining the causes behind specific cases of lead poisoning but also of ensuring that lead sources are eliminated.

Unlike most water contaminants, lead gets into water after it leaves a water treatment plant. Often this contamination is the result of water treatment changes meant to improve water quality that end up altering the water chemistry, destabilizing lead-bearing mineral scales that coat service lines and corroding lead solder, pipes, faucets, and fixtures. “Lead is a ‘close-to-home’ contaminant,” says Marc Edwards, an environmental engineer at Virginia Polytechnic Institute and State University. “That makes it very difficult to regulate and monitor.”

Under the U.S. Environmental Protection Agency’s (EPA) 1991 Lead and Copper Rule (LCR), municipal water utilities must sample a small number of homes at high risk for elevated lead levels, such as those known to have lead plumbing components. The size of the water system determines how many samples must be collected in each sampling period (the maximum required is 100), and the sampling interval can vary from 6 months to 3 years, depending on past compliance. The law requires that samples be “first-flush” water that has stood in pipes for a minimum of 6 hours. This scenario represents high but routine exposures to lead in tap water, because the longer corrosive water sits in contact with lead parts, the more lead leaches out. In many households, this worst-case normal-use scenario happens twice daily Monday through Friday: in the morning when the residents awake, and in the afternoon when they return home from work and school.

Under the LCR, utilities are required to notify customers and take remedial action if more than 10% of the households sampled have tap water with lead levels exceeding 15 ppb. Remedial action might include changing chemical treatment methods to make the water less corrosive or, if treatment fails, to replace lead pipes that lie beneath publicly owned spaces such as streets and sidewalks. These provisions would seem to suggest that if a water utility is in compliance with the rule, then none of the dwellings served by the utility need worry about their tap water being a significant source of lead. Yet LCR compliance is based upon the results of sampling only a tiny percentage of the homes served. So even when a utility is entirely within LCR compliance, some consumers may unknowingly receive and consume water that contains lead levels much higher than 15 ppb.

New evidence linking low-level lead exposure with cognitive deficits and other data linking lead-contaminated water with increases in the prevalence of children having blood lead levels over 10 μg/dL suggest that testing water for lead contamination should be done routinely in older cities.

—Bruce Lanphear, Simon Fraser University

“EPA as the regulator of lead in tap water and CDC [Centers for Disease Control and Prevention] with its concern for preventing lead poisoning in children should be working together to get on top of this problem,” says Edwards. “But in my experience this is not occurring to the extent it should.”

## Providence

Rhode Island has one of the country’s most serious lead problems. According to the Rhode Island Department of Health, the state has 3 times the U.S. average number of children with blood lead levels above 10 μg/dL, the “level of concern” at which the CDC recommends intervention. The state, like most of New England, also has soft, naturally corrosive water and, with the fifth-oldest housing stock in the nation, tens of thousands of lead water pipes are still in use. In Providence alone, about 27,000 homes (or 27% of the total housing stock) have lead service lines, according to Pamela Marchand, head of the city’s Water Supply Board. Despite numerous attempts to control lead corrosion by modifying the water chemistry, the utility has consistently failed to meet LCR action levels and in 2006 began replacing the publicly owned portion of the lead pipes for about 1,000 homes per year.

At the same time, roughly 10% of Providence children with blood lead levels high enough to require a home inspection also have high lead levels in their tap water, confirms Rhode Island Department of Health spokeswoman Annemarie Beardsworth. From September 2003 to March 2007, the state conducted over 300 home inspections for children with elevated blood lead levels—meaning 1 blood lead test result of at least 20 μg/dL or 2 test results of at least 15 μg/dL taken between 3 and 12 months apart. Samples exceeded the EPA action level of 15 ppb in 37 of the inspections, with a maximum measured concentration of 152 ppb.

Compared with many other states, Rhode Island is unusually proactive in routinely collecting water samples during such home inspections. Yet standard Rhode Island protocol is to collect samples after 1 minute of flushing the target tap, which reflects a remedial measure that the health department recommends to a family after a child with elevated blood lead levels is identified. And taking samples after flushing can result in lead levels that are lower than those in unflushed samples.

Michael Moore, director of the Australian National Research Centre for Environmental Toxicology at the University of Queensland, says, “I am very surprised to see such high concentrations in flushed samples. Usually flushing drops the lead dramatically. In our work, fully flushed samples have lead concentrations ten times lower than first-flush samples. These levels of lead in the water could certainly cause the high blood lead levels in these children.” Moore’s work in the 1970s was key in revealing the link between elevated blood lead levels in children in Glasgow, Scotland, and high levels of lead at the tap.

The Rhode Island Department of Health contends that water is not a primary source of lead exposure to children in any of these cases, according to Beardsworth. She points out that in Providence, both the incidence and prevalence of lead poisoning have dramatically decreased—from 11.7% and 19.8%, respectively, in 1998 to 2.7% and 4.2% in 2007—while the levels of lead in Providence tap water have stayed the same or increased slightly. “Our opinion is that other sources are responsible [for elevated blood lead],” she says—even in cases where flushed samples collected at random during the day exceed 100 ppb. “The health department generally finds that lead hazards from paint, dust, and soil are the primary sources of exposure for a child with significant lead poisoning,” Beardsworth says.

But Bruce Lanphear, a pediatric epidemiologist at Simon Fraser University in Vancouver who has studied lead effects on children, is not as certain. He says many, if not most, urban children with blood lead levels greater than 10 μg/dL have multiple sources of lead exposure, including water. “New evidence linking low-level lead exposure with cognitive deficits and other data linking lead-contaminated water with increases in the prevalence of children having blood lead levels over ten micrograms per deciliter suggest that testing water for lead contamination should be done routinely in older cities,” he says.

## Portland

Portland, Oregon, takes a unique approach to addressing lead in water, an approach that has won both accolades and accusations. “Portland has very interesting water politics and dynamics, and people did not want to add chemicals that most utilities usually use for corrosion control,” Lisa Ragain, a Portland risk communications consultant, explained at the American Public Health Association (APHA) annual meeting in Philadelphia in November 2009. (Indeed Portland is also the largest U.S. city that does not fluoridate its drinking water, and it is one of the few major cities that does not filter its water.) The city has opted instead for partial corrosion control combined with aggressive public education aimed at lead paint abatement.

The city has no lead water pipes, but thanks to water that is naturally very corrosive, lead may leach from solder and brass plumbing that can be labeled “lead free” but still contain up to 8% lead. Since 2000 the city has exceeded the LCR action level 5 times, most recently in 2006, according to Oregon state records. In 2005 the Portland Water Bureau implemented partial corrosion control that program manager Scott Bradway says has reduced lead in water levels by more than 75%.

Compliance with the LCR could be achieved with optimized corrosion control similar to what many other cities use, according to Ragain’s presentation. But based on the city’s preference for minimal water treatment and the belief that paint is a more significant source of lead to children than water, Portland instead spends $500,000 annually on a public education campaign and lead paint abatement program. “This approach was a win–win for community public health, reducing lead exposure across the community and across media of exposure, especially for children,” says David Leland, manager of the Oregon Department of Human Services Drinking Water Program.

Water regulators have divergent attitudes toward Portland’s approach. The state regulators are enthusiastic about the program. “Look at the hierarchy of concern for lead,” says Leland. “Number one was the lead from gasoline in the air, before it was banned. Now it’s paint,” he says. The 2004 Government Accountability Office report *Drinking Water: Safeguarding the District of Columbia’s Supplies and Applying Lessons Learned to Other Systems* praised Portland’s multimedia approach, in particular pointing to the city’s effective methods for notifying residents about problems.

But Harold Rogers, EPA Region 10 Safe Drinking Water Act coordinator, notes that many Portland residents likely are being exposed to lead in drinking water without their knowledge. “[Portland does] mainly do lead education—that’s a good thing, but it’s not so good for people who unwittingly have high levels of lead at the tap,” he says. The Portland Water Bureau offers free lead-in-water testing upon request, and the bureau’s data on this testing give an indication of the problem mentioned by Rogers. Since 2006, 3,205 tap water samples taken by the city of Portland have been tested. Twenty-five samples of every 1,000 have measured over 15 ppb, 1 of every 100 has measured over 35 ppb, and 1 of every 1,000 has measured over 120 ppb. The highest sample, taken in August 2008, measured 910 ppb. These self-selected homes are not from the high-risk compliance sampling pool.

EPA headquarters holds a similar view to Rogers. “Portland Water Bureau has not exceeded the lead action level since December 2006, and the system performs extensive public outreach to educate the public about possible exposure. However, without conducting optimal corrosion control, they are still in violation of the treatment technique requirements of the Lead and Copper Rule,” says EPA spokeswoman Enesta Jones. Portland can be simultaneously in and out of compliance thanks to a loophole in the LCR that allows the primary regulator, usually the state health or environmental protection department, to independently define “optimal corrosion control” and thus allow flexibility in water lead concentrations in order to meet other drinking water laws, according to EPA insiders.

Another loophole that relates to which homes are sampled for compliance monitoring may also be fostering a picture of Portland’s water that is rosier than reality. Between September 2000 and November 2001, three rounds of compliance monitoring at 100 high-risk homes showed that at least 10% of the samples exceeded the 15-ppb action level. Compliance monitoring from 2002 onward showed generally lower levels of lead in tap water, an achievement the water bureau credits to higher pH levels in the water. But the lower levels also coincided with a change in the 100 high-risk homes selected for compliance monitoring, according to a 5 October 2004 *Washington Post* investigative report: In 2002, “the utility dropped more than half the homes with lead higher than the federal limit, replacing them with suburban homes that had, on average, significantly lower levels, [state] records show.”

Such a change in sampling “goes against the spirit of the LCR,” says Jim Elder, who headed the EPA drinking water program from 1991 to 1995. “The monitoring is a dynamic protocol for sampling that is supposed to reflect constant vigilance—going after the homes at risk. If you know that tap water lead is high in the city, then that’s where you should look.” In addition, Portland’s choice between optimum corrosion control and public education is a “covert form of cap and trade,” Elder says.

## A Problem Not Unknown

In her presentation at the November 2009 APHA annual meeting, Virginia Tech environmental engineer Simoni Triantafyllidou noted that until about 1985 water was generally acknowledged as potentially a significant source of lead exposure. Prior to this time, many studies demonstrated a strong correlation between lead in water and children’s blood lead levels. The impact of lead in commercial infant formula that was marketed in 1975 and 1976 on blood lead levels was determined by Jacqueline E. Ryu et al. They reported in the September 1983 issue of the *American Journal of Diseases of Children* that infants who were fed formula with 70 ppb lead had blood lead levels that spiked to an average of 14.4 μg/dL within a few months. When the formula contained 10 ppb lead, the children’s blood lead was stable at an average of 7.2 μg/dL. In 1985, R. F. Lacey et al. reported in the 1 March 1985 issue of *Science of the Total Environment* that a 100-ppb increase in water lead levels resulted in an average increase in children’s blood lead of 6.2 μg/dL.

According to Triantafyllidou, the public health mindset in the United States appears to have changed in the mid 1980s with the onset of studies such as the Cincinnati Lead Study, a long-term research effort that helped put lead paint and dust front and center in the struggle to reduce children’s exposure to lead. Despite its many successes, the study failed to adequately account for water, she says. “The researchers did not measure lead in water at all as part of their study. Instead, they cited a contact from the water utility, saying that lead in water samples from the distribution system had measured to be very low with a median lead concentration below the detection limit of five parts per billion,” she says. “Perhaps tap water would have measured very low in lead if they did check. But they did not check, and we know that samples from the distribution system are not necessarily representative of exposure at the tap.”

Nonetheless, a few studies continued to consider tap water as a source of lead exposure. For instance, Lanphear et al. noted in the February 1998 issue of *Environmental Research* that blood lead levels correlated with higher water lead even in situations where a citywide problem with water lead was not recognized. Since 2004 drinking water has been directly linked to elevated levels of lead in children’s blood in Washington, DC, North Carolina, and Maine [see “Out of Plumb: When Water Treatment Causes Lead Contamination,” *EHP* 117:A542–A547 (2009)]. A February 2007 *EHP* research article by Marie Lynn Miranda and colleagues documented that changes in water treatment also have been linked with broad increases in children’s blood lead levels. As a result, more experts believe the problem of lead in drinking water is much bigger than currently recognized.

“The problem is that water is in everyone’s home,” Moore explains. “Even if people don’t drink tap water, they cook with it. Lead slams straight into pasta. Boil up peas in contaminated water, and the lead is in the peas.”

Mary Jean Brown, chief of the CDC Lead Poisoning Prevention Branch, says all sources of lead are important to consider, especially when it comes to children’s exposure. “Individuals may have legitimate differences of opinion about the relative contribution of drinking water lead and be in total agreement about the need to remove this source of exposure,” she says. “It would be a mistake to place various sources of lead in competition with each other. Identifying and removing sources of lead before children are exposed should be our focus.”

Yet the majority public health opinion in the United States remains largely blind to water as a source of lead to children, according to Ralph Scott, former community projects director at the Alliance for Healthy Homes, who described the current situation at the National Environmental Public Health conference in Atlanta, Georgia, in October 2009. The confusion begins with questions of how—or even if—to sample for lead in water in the homes of children with elevated blood lead levels.

## Confusion about Sampling

The CDC and the EPA do not provide specific guidance on when and how to test water for lead. Health agencies wanting to address lead at the tap are largely on their own, says Scott, who notes that no government agency currently identifies a specific threshold amount of lead in water as a hazard. Prior to 2004, the EPA Office of Water provided the most specific information, advising that “lead at concentrations of 40 ppb or higher poses an imminent and substantial endangerment to the health of children and pregnant women.” But in March 2004, the EPA removed this statement from its website. “When EPA updated its website, the agency found there was no reference for that risk estimate and found no research on which it was based,” says Jones.

As a result, current official recommendations for assessing the risk of lead exposure typically omit or downplay water, says Scott. For instance, the 2002 CDC document *Managing Elevated Blood Lead Levels Among Young Children: Recommendations from the Advisory Committee on Childhood Lead Poisoning Prevention* notes the water supply should be considered only “when no other source of lead is found.”

When a child has elevated blood lead levels, the CDC Lead Poisoning Prevention Program’s state and local grantees are often the groups that direct home inspections designed to locate the source of the lead. An estimated 30% or more of cases of elevated blood lead do not have an immediate lead paint source, and no source at all can be identified for 5–10% of cases, according to a review by Ronnie Levin et al. in the October 2008 issue of *EHP*. Even in cases where lead dust is implicated and remediated, blood lead levels often fail to fall, Brown and colleagues reported in the January 2006 issue of *Pediatrics*. “The distribution of lead in the body is one plausible explanation of why blood lead levels in children do not decline rapidly,” Brown says. She points to a study by Roberto Gwiazda et al. in the January 2005 issue of *EHP* that found that bone acted as an endogenous source of lead after home remediation, contributing as much as 96% of blood lead in the children studied.

The problem is that water is in everyone’s home. Even if people don’t drink tap water, they cook with it. Lead slams straight into pasta. Boil up peas in contaminated water, and the lead is in the peas.

—Michael Moore, University of Queensland

According to Lanphear, another reason for the lack of reduction may be that the current safety standard for dust lead is so lenient that even a remediated home is still hazardous. Edwards agrees with Lanphear for many cases, but also believes still another reason children’s blood lead levels could fail to fall is that the remediation ignored water as an ongoing important background source of lead exposure. With a team of undergraduate students, in 2006 Edwards surveyed a group of state and local agencies on the front line of lead poisoning prevention to find out how they deal with the potential of lead exposure via drinking water. He presented the results at the November 2009 APHA meeting.

The Virginia Tech students contacted agencies in 21 cities and states and received 17 responses. They found that 2 states, Connecticut and North Carolina, require water tests during home inspections of children with elevated blood lead levels. Agency staff in Arizona, Los Angeles, and Iowa told the students that they “often” test. Staff in Florida, Kansas, Massachusetts, New York, Nevada, Ohio, Texas, and Wisconsin said they “sometimes” test, and respondents from Detroit, Oklahoma, Philadelphia, and Washington, DC, said they “never” test.

Comments made in the 2006 survey by the 8 jurisdictions that sometimes test indicated they do so very infrequently. For example, Florida officials test only if water is a suspected source. In Kansas, the water is tested if no other lead sources are found and if lead plumbing is known to exist. Massachusetts officials test the water only if a child’s elevated blood lead persists after paint hazards have been addressed or if requested by the occupants. If a municipality in Nevada exceeds the 15-ppb action level, then home inspectors test. Ohio inspectors will test the water if it is a suspected source and if it is from a private well or other private supply.

The Virginia Tech survey responses also revealed there is no standard protocol for sampling the water in the home of a child with elevated blood lead. If a water sample is taken at all, it tends to be obtained at the time of the inspection, in whatever way the inspector chooses to sample. This means that in the few instances when agencies do collect water, they usually do not collect samples with the high levels that normal use patterns in the United States can produce, and that are needed to characterize risk, says Levin. Moreover, says EPA corrosion chemist Michael Schock, “Not only are [inspectors] not collecting a well-defined sample—representing any particular kind of exposure scenario, right or wrong—they are haphazardly sampling in a way that defeats any ability to make comparisons to other sites, within or outside of their particular investigation. So, there is a big loss of potentially useful information on lead exposure sources and amounts to public health agencies across the United States.”

## Potential for Change

Since October 2008, the EPA has been considering the possibility of long-term revisions to the LCR, according to Jeff Kempic, treatment technology and cost team leader with the EPA Office of Ground Water and Drinking Water. Among the topics being considered are sampling protocols, how utilities should determine the most at-risk housing, and whether replacing the publicly owned portion of lead water lines is beneficial. The agency is making progress, but there is no deadline for these revisions, he says.

“It is very hard to persuade cash-strapped organizations trying to protect children that they need to spend money on water testing,” says Scott. But reluctant municipalities might look to Washington, DC, which presents a good example of how the official attitude toward drinking water as a potential lead exposure source can change, says Scott. The city, which prior to 2006 rarely if ever tested drinking water in properties associated with elevated blood lead in children, now does so routinely—and inspectors are finding some high levels. An educational fact sheet is being written by the DC Department of the Environment with input from community health advocates for parents and guardians, and the city is considering legislation that would include a ban on lead plumbing fixtures and possibly other measures to address lead levels in drinking water.

Such efforts may be especially important given the realities of human nature. Cities with a problem of lead in their water often advise residents to flush their water before drinking or cooking—sometimes for as long as a minute—and to never use hot water tap for food preparation. “Of course it’s not realistic to assume many people will follow such a recommendation,” says Scott. “Even if someone decides that flushing is a good idea, sixty seconds seems like forever, and even many conscientious people will grow impatient and cut the flushing short.” In fact, in a study by Regina Fertmann et al. reported in the July 2004 issue of the *International Journal of Hygiene and Environmental Health*, although flushing lowered the blood lead levels in German women whose tap water contained at least 5 ppb lead, the majority of subjects considered flushing to be an unsustainable health preventative behavior in the long term. It’s also difficult for many people, particularly children, to judge time when flushing.

“This entire issue of water as a source of lead for children is surrounded by assumptions that could well be masking a significant problem,” says Scott. “The exposure pathway is clear—from the plumbing to the tap to the child—but [lead-]contaminated water looks, smells, and tastes exactly like pure water. The only way to know is to measure lead levels accurately. But we aren’t. It’s a sure bet you won’t find something if you don’t look for it.”

## Lead Absorption and Storage in the Human Body

The human body cannot use lead but will absorb and store it in various tissues, predominantly the bones and teeth; lead also circulates in the blood. People can excrete a certain amount of the lead they breathe or swallow. Efficiency of excretion depends on age.

Infants and young children are believed to absorb about 40–50% of ingested water-soluble lead; adults absorb 3–10%, but this amount may increase to 50–60% during fasting. Studies with stable lead isotopes raise some uncertainty about the difference in estimates of gastrointestinal absorption between children and adults.

If more lead is absorbed than is excreted, obviously the body burden increases. Stored lead can be released back into the blood stream during events marked by bone turnover, such as pregnancy, menopause, and bone breaks.

## Several factors appear to increase the amount of lead that is absorbed and stored

- A diet deficient in calcium, iron, and/or zinc
- Being in a state of increased calcium demand, such as during pregnancy and lactation
- Genetic factors that affect the efficiency of iron or calcium absorption
- Exposure to cigarette smoke
- Lead ingestion on an empty stomach

Source: U.S. EPA. 2006. Air quality criteria for lead. Washington, DC: U.S. Environmental Protection Agency), National Center for Environmental Assessment, Office of Research and Development.

## Figures and Tables

**Figure:**
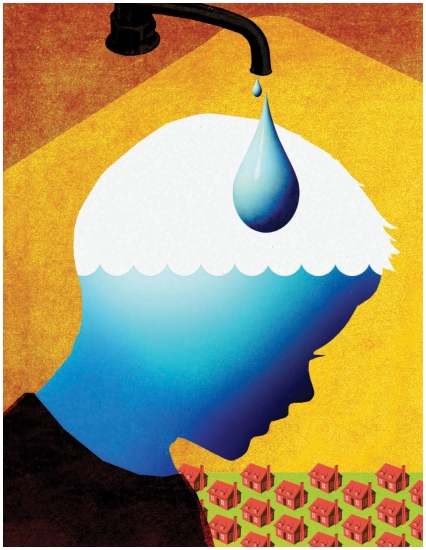


**Figure:**
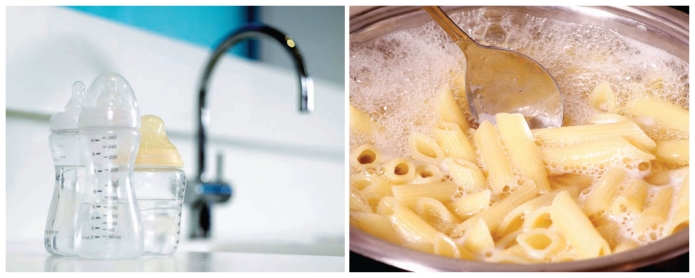
The way people use water can influence how much lead exposure they receive. Sometimes when there is a problem with lead in tap water, public health officials may recommend letting the tap run for at least a minute before drawing water and using only cold water for formula preparation and cooking. But a minute can seem like an eternity when it’s time to eat, and it can be hard to judge time. So even conscientious consumers may end up preparing food using lead-heavy first-flush water.

**Figure:**
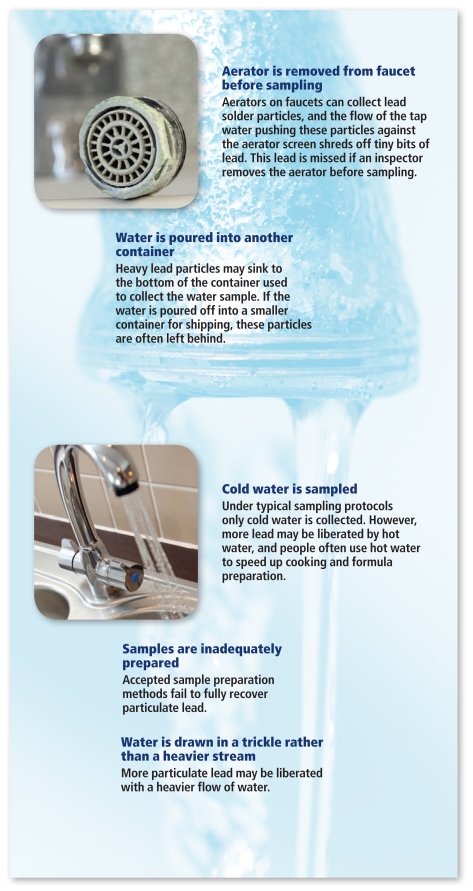
How Sampling Can Overlook Lead in Water Source: Edwards M, et al. 2009. Lead in drinking water: sampling variability and analytical issues. Presented at: APHA Annual Meeting, Philadelphia, PA; 7–11 November 2009.

**Figure:**